# Profiling and Pharmacokinetic Studies of Alkaloids in Rats After Oral Administration of *Zanthoxylum nitidum* Decoction by UPLC-Q-TOF-MS/MS and HPLC-MS/MS

**DOI:** 10.3390/molecules24030585

**Published:** 2019-02-07

**Authors:** Aihua Huang, Yuguang Chi, Jiawei Liu, Mincun Wang, Jialiang Qin, Lijuan Ou, Weiwen Chen, Zhongxiang Zhao, Ruoting Zhan, Hui Xu

**Affiliations:** 1 Key Laboratory of Ministry of Education, Research Center of Chinese Herbal Resources and Engineering, Guangzhou University of Chinese Medicine, Guangzhou 510006, China; hah2008@gzucm.edu.cn (A.H.); liujw@gzucm.edu.cn (J.L.); liang8133466@163.com (J.Q.); chenww@gzucm.edu.cn (W.C.); 2 School of Pharmaceutical Sciences, Guangzhou University of Chinese Medicine, Guangzhou 510006, China; ygchi@gzucm.edu.cn (Y.C.); 13312587641@163.com (M.W.); oulijuan@gzucm.edu.cn (L.O.); zzx37@163.com (Z.Z.)

**Keywords:** *Zanthoxylum nitidum*, UPLC-Q-TOF-MS/MS, pharmacokinetic study, HPLC-MS/MS, magnoflorine

## Abstract

*Zanthoxylum nitidum* (Roxb.) DC (Rutaceae), called as “liangmianzhen” in China, is well known for its anti-inflammation and analgesic effect. Alkaloids are its main active constituents. However, little has been known about the absorption of main alkaloids in vivo. In this study, an ultra-performance liquid chromatography coupled with quadrupole-time-of-flight mass spectrometry was employed for identification of absorbed alkaloids in rats after oral administration of *Z. nitidum* decoction. By analyzing the fragmentation patterns, a total of nineteen alkaloids were exactly or tentatively identified in rat plasma after treatment, of which magnoflorine, α-allocryptopine, and skimmianine are dominant. Moreover, a high performance liquid chromatography coupled mass spectrometry method was developed for simultaneous quantification of magnoflorine, α-allocryptopine, and skimmianine, and successfully applied to pharmacokinetic study in rats after oral administration of *Z. nitidum* decoction. The research would contribute to comprehensive understanding of the material basis and function mechanism of *Z. nitidum* decoction.

## 1. Introduction

*Zanthoxylum nitidum* (Roxb.) DC (Rutaceae), locally called as “liangmianzhen” belongs to the genus Zanthoxylum of family Rutaceae. Its roots are traditionally used for treating various ailments such as toothache, stomachache, fever, rheumatism, paresis, and boils, and can be used as an insecticide [1]. Our previous studies indicated that *Z. nitidum* decoction has anti-contusion injury, analgesic, anti-inflammation, anti-gastritis, gastric mucosal protection, and gastrointestinal movement promotion effects [2,3]. Alkaloids are proved to be the major bioactive components of *Z. nitidum* [4,5,6,7,8,9,10,11,12,13,14,15,16,17,18,19,20]. Recently, alkaloid profiling of *Z. nitidum* by HPLC-Q-TOF-MS had been reported [21,22]. Until now, up to 50 alkaloids were isolated and identified, which mainly belong to aporphine, benzylisoquinoline, protoberberine, protopine, benzophenanthrindine, and quinoline alkaloids (Table S1). Our study gave similar results. On the other hand, it is generally accepted that only the components absorbed in blood might contribute to the therapeutic effects. Despite of critical pharmacological function of *Z. nitidum* decoction, its absorbed alkaloid profile as well as pharmacokinetic behavior in vivo remain unknown. Moreover, in order to understand the material basis and function mechanism of *Z. nitidum* decoction, it is necessary to depict the absorption and pharmacokinetics of the major bioactive alkaloids in vivo after oral administration.

Ultra-performance liquid chromatography coupled with quadrupole-time-of-flight mass spectrometry (UPLC-Q-TOF-MS/MS) has been widely applied for characterization of the components in Chinese medicines and prescriptions [23,24]. Owing to its high resolution and sensitivity, UPLC-Q-TOF-MS/MS can provide a simple and efficient approach for speculating unknown components besides identifying the known ones [25]. In this paper, the absorbed alkaloids in vivo were analyzed by UPLC-Q-TOF-MS/MS. Based on the fragmentation patterns of five authentic alkaloids and those reported in literatures, nineteen alkaloids were exactly identified or tentatively identified in rat plasma after oral administration of *Z. nitidum* decoction. Five of them were reported for the first time in *Z. nitidum*. Meanwhile, pharmacokinetic behavior of *Z. nitidum* decoction was further investigated by HPLC-MS/MS for the first time. The study would provide key information for understanding of the function mechanism of *Z. nitidum* decoction as well as quality control.

## 2. Results and Discussion 

### 2.1. Identification of Absorbed Alkaloids of Z. nitidum Decocotion in Rat Plasma

**Figure 1:**
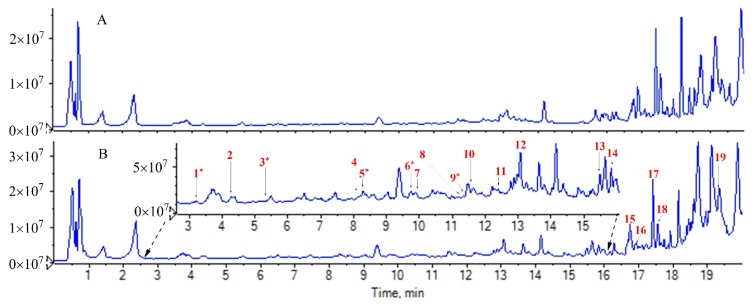
TIC chromatograms. (**A**) Blank plasma; (**B**) plasma after oral administration of *Z. nitidum* decoction.

**Figure 2:**
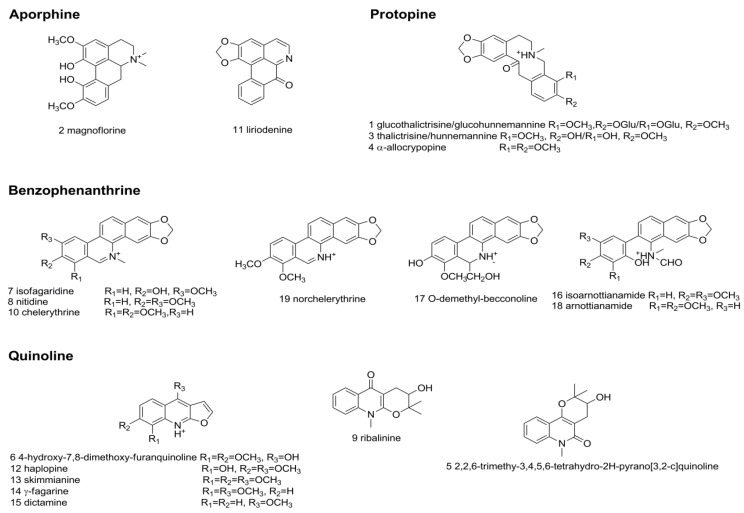
Chemical structure of the alkaloids in rat plasma after oral administration of *Z. nitidum* decoction.

To identify the absorbed alkaloids *in vivo*, the rat plasma after oral administration of *Z. nitidum* decotion was analyzed using the target (Table S1) and untarget strategy reported by Zhang et al. [26]. As a result, a total of 19 prototype alkaloids were identified, including 2 aporphinoid, 3 protopine, 7 benzophenanthrindine, and 7 quinoline alkaloids (Table 1). Among 19 compounds, magnoflorine, α-allocryptopine, nitdine, chelerythrine, and skimmianine were unambiguously characterized by comparison with authentic standards. Other compounds were tentatively deduced based on accurate mass of quasimolecular, MS^2^ spectra and fragmentation pathway, and some isomers were further differentiated by considering relative retention time and molecular polarity. The total ion chromatograms (TICs) of these components are shown in Figure 1 and their chemical structures are shown in Figure 2. The extract ion chromatograms (EICs) and MS^2^ spectra are given in Figure S1. 

**Figure 3:**
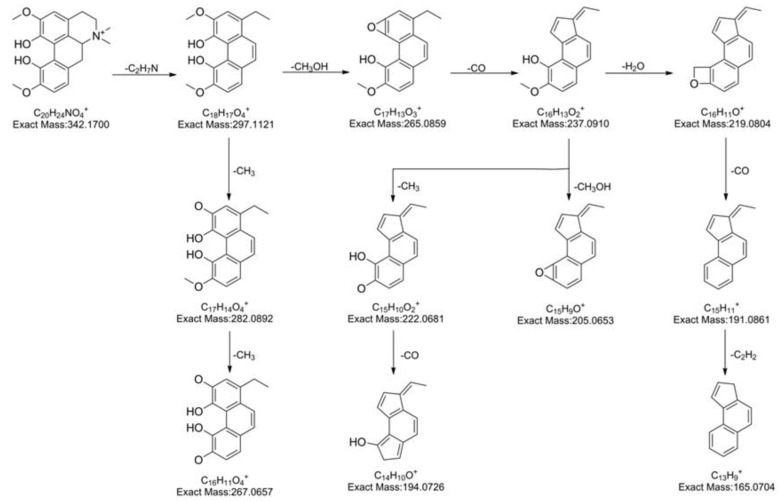
The proposed fragmentation pathway of magnoflorine.

**Figure 4:**
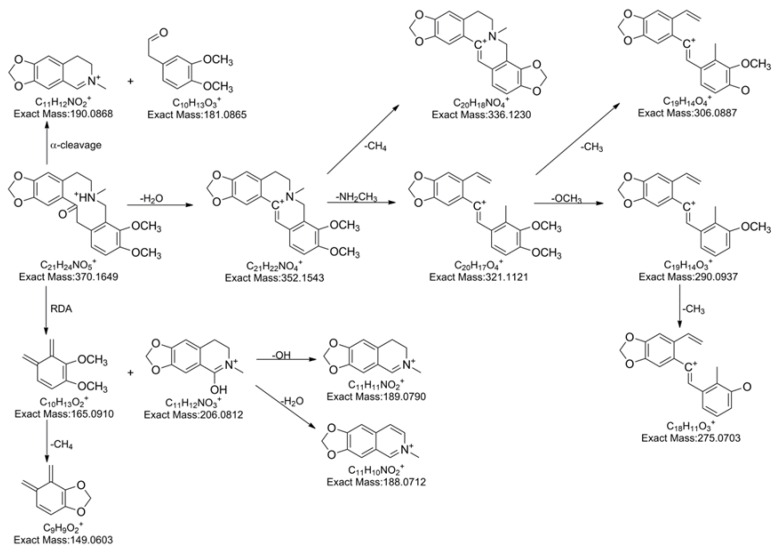
The proposed fragmentation pathway of α-allocryptopine.

**Figure 5:**
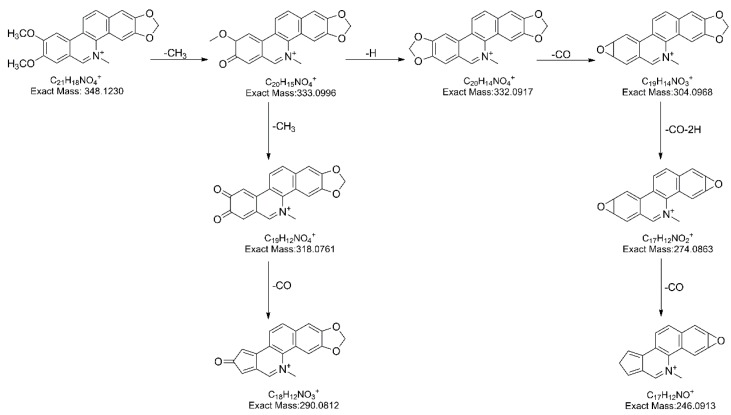
The proposed fragmentation pathway of nitidine.

To facilitate alkaloid identification, **5** authentic standards representing **5** known alkaloids in *Z. nitidum*, including one aporphine (magnoflorine), one protopine (α-allocryptopine), two benzophenanthrindine (nitidine and chelerythrine), and one quinoine (skimmianine) were selected and analyzed thoroughly to illustrate the proposed fragmentation pathways for references (Figure 3, Figure 4 and Figure 5). Among them, the fragment pathway of skimmianine is given in our previous published reference [27].

Magnoflorine was eluted at 4.2 min with the parent ion at *m*/*z* 342.1706 (C_20_H_24_NO_4_^+^). The fragment ion at *m*/*z* 297.1121 (C_18_H_17_O_4_^+^) was attributed to the elimination of (CH_3_)_2_NH, which might be an important characteristic of aporphine alkaloid fragmentation pathway [28]. Subsequently, the fragment ion at *m*/*z* 265.0859 (C_17_H_13_O_3_^+^) was observed as the base peak due to the loss of CH_3_OH. Because of the electron-withdraw inductive effect and the minimal energy of ion, the expulsion of CH_3_OH could occur from vicinal hydroxyl and methoxy groups on C1 and C2 [29]. The fragment ion at *m*/*z* 237.0910 (C_16_H_13_O_2_^+^) was produced by the neutral loss of CO from the fragment ion at *m*/*z* 265.0859 (C_17_H_13_O_3_^+^). The removal of CH_3_OH followed by CO in vicinal hydroxyl and methoxy groups is an important fragmentation pathway of aporphine alkaloids. The fragment ion at *m*/*z* 237.0910 (C_16_H_13_O_2_^+^) further fragmented in three ways. The first way yielded fragment ions at *m*/*z* 219.0804 (C_16_H_11_O^+^), 191.0861 (C_15_H_11_^+^), 165.0704 (C_13_H_9_^+^) after the consecutive loss of H_2_O, CO, and C_2_H_2_. The second produced fragment ions at *m*/*z* 222.0681 (C_15_H_10_O_2_^+^), 194.0726 (C_14_H_10_O^+^) by the successive elimination of CH_3_ and CO. While the ion at *m*/*z* 205.0853 (C_15_H_9_O^+^) was the result of the loss of CH_3_OH through the third way. The fragment ion at *m*/*z* 297.1121 (C_18_H_17_O_4_^+^) might break up into the ions at *m*/*z* 282.0892 (C_17_H_14_O_4_^+^), 267.0657 (C_16_H_11_O_4_^+^) via the consecutive loss of CH_3_ and CH_3_.

α-allocryptopine gave parent ion at *m*/*z* 370.1651 (C_21_H_24_NO_5_^+^) at 8.1 min. Subsequently, the parent ion fragmented in three main ways. The first way generated fragment ions at *m*/*z* 206.0811 (C_11_H_12_NO_3_^+^), 165.0916 (C_10_H_13_O_2_^+^) by retro-Diels-Alder (RDA) reaction. The ion at *m*/*z* 206.0811 (C_11_H_12_NO_3_^+^) further fragmented in two ways, generating fragment ions at *m*/*z* 189.0780 (C_11_H_11_NO_2_^+^) and 188.0704 (C_11_H_10_NO_2_^+^). The loss of OH led to the formation of ion at *m*/*z* 189.0780 (C_11_H_11_NO_2_^+^), while the ion at *m*/*z* 188.0704 (C_11_H_10_NO_2_^+^) was produced via the loss of H_2_O. The ions at *m*/*z* 206.0811 (C_11_H_12_NO_3_^+^), 189.0780 (C_11_H_11_NO_2_^+^) and 188.0704 (C_11_H_10_NO_2_^+^) were the dominant peaks in the MS^2^ spectrum of α-allocryptopine, indicating that the loss of H_2_O or OH following the RDA reaction must be the major fragmentation pathway. Concerning the second fragmentation way, the parent ion might undergo α-cleavage, producing the ions at *m*/*z* 181.0865 (C_10_H_13_O_3_^+^). Otherwise, the parent ion might break up into the ions at *m*/*z* 352.1543 (C_21_H_22_NO_4_^+^), 321.1124 (C_20_H_17_O_4_^+^), 290.0937 (C_19_H_14_O_3_^+^) and 275.0703 (C_18_H_11_O_3_^+^) after the consecutive loss of H_2_O, NH_2_CH_3_, OCH_3_ and CH_3_. The minor peaks at *m*/*z* 336.1230 (C_20_H_18_NO_4_^+^) and 306.09.4 (C_19_H_14_O_4_^+^) might be produced by the loss of CH_4_ from the ion at *m*/*z* 352.1543 (C_21_H_22_NO_4_^+^) and by the loss of CH_3_ from the ion at *m*/*z* 321.1124 (C_20_H_17_O_4_^+^), respectively. The loss of H_2_O or OH following the RDA reaction, as well as the loss of H_2_O, were notable fragmentation pattern of α-allocryptopine. This notable fragmentation pattern is consistent with the published fragmentation of protopine-type alkaloids [30].

Two isomers, nitidine and chelerythrine at retention time 11.3 and 11.6 min, respectively, both showed parent ion at *m*/*z* 348.1236 (C_21_H_18_NO_4_^+^) and similar fragment pathway. Due to its vicinal methoxy groups, the fragment ion at *m*/*z* 333.0996 (C_20_H_15_NO_4_^+^), 332.0917 (C_20_H_14_NO_4_^+^), 304.0968 (C_19_H_14_NO_3_^+^), 274.0863 (C_18_H_12_NO_2_^+^) and 246.0913 (C_17_H_12_NO^+^) were observed as a result of the elimination of CH_3_, H, CO, CH_2_O and CO. Another route produced 333.0996 (C_20_H_15_NO_4_^+^), 318.0761 (C_19_H_12_NO_4_^+^), and 290.0812 (C_18_H_12_NO_3_^+^) via the successive loss CH_3_, CH_3_ and CO.

Thus, compounds (**2**, **4**, **8**, **10**, **13**) were unambiguously identified as magnoflorine, α-allocryptopine, nitidine, chelerythrine, and skimmianine, respectively.

Compound **11** displayed the protonated molecule ion at *m*/*z* 276.0655 (C_17_H_10_NO_3_^+^) at retention time of 12.2 min. It produced the fragment ions at *m*/*z* 248.0706 (C_16_H_10_NO_2_^+^), 218.0600 (C_15_H_8_NO^+^) and 190.0651 (C_14_H_8_N^+^) via the successive loss of CO, CH_2_O and CO. By comparing with the data in literature, compounds **11** was tentatively identified as liriodenine [5].

Besides α-allocryptopine, two other protopine alkaloids (**1**, **3**) were observed. Compounds **1** and **3** were reported for the first time in *Z. nitidum*. These two compounds were following notable fragmentation pathway: the loss of H_2_O or OH following the RDA reaction and the loss of H_2_O from parent ion.

Compound **3** exhibited the protonated molecule ion at *m*/*z* 356.1498 (C_20_H_22_NO_5_^+^) at retention time of 5.3 min, 14 u less than that of α-allocryptopine. The fragment ions at *m*/*z* 206.0811 (C_11_H_12_NO_3_^+^), 189.0780 (C_11_H_11_NO_2_^+^) and 188.0704 (C_11_H_10_NO_2_^+^) resulted from the characteristic losses of H_2_O or OH following the RDA reaction. The fragment ion at *m*/*z* 338.1389 (C_20_H_20_NO_4_^+^) was the result of the loss of H_2_O. Compounds **3** was identified as hunnemannine or thalictrisine [31]. Compound **1** eluted at 3.2 min, had molecular formula (C_26_H_32_NO_10_^+^), 162 u more than that of compound **3**. The main fragment ions were identical to those of compound **3**. Compound **1** was speculated to be glucothalictrisine or glucohunnemannine.

In addition to nitidine and chelerythrine, five benzophenanthridine alkaloids (**7**, **16**, **17**, **18**, **19**) were observed. Benzophenanthrindine alkaloids strictly keep their nitrogen inside the highly aromatic ring during fragmentation. The fragment ions were mainly produced by the peripheral loss from methoxyl, methylenedioxy and N-CH_3_ groups and so on.

Compound **7** exhibited molecule ion at *m*/*z* 334.1079 (C_20_H_16_NO_4_^+^) at retention times of 9.9 min. It produced the fragment ions at *m*/*z* 319.0839 (C_19_H_13_NO_4_^+^), 291.0890 (C_18_H_13_NO_3_^+^) and 276.0655 (C_17_H_10_NO_3_^+^) via the successive loss of CH_3_, CO and CH_3_. Compound **7** was identified as isofagaridine after compared with data in the literature [14].

Eluted at 19.1 min, compound **19** showed the protonated molecular ion at *m*/*z* 334.1074 (C_20_H_16_NO_4_^+^). It produced the fragment ions at 319.0839 (C_19_H_13_NO_4_^+^), 318.0761 (C_19_H_12_NO_4_^+^) and 290.0812 (C_18_H_12_NO_3_^+^) due to the successive loss of CH_3_, H and CO, suggesting that compound **19** contained vicinal methoxy groups. Compound **19** was identified as norchelerythrine after compared with the literature [6].

Compound **17** was eluted at 17.4 min, and displayed the protonated molecule ion at *m*/*z* 366.1336 (C_21_H_20_NO_5_^+^). It produced the fragment ions at *m*/*z* 348.1220 (C_21_H_18_NO_4_^+^), 320.0917 (C_19_H_14_NO_4_^+^) via the successive loss of H_2_O and C_2_H_4_ on C8 and N, respectively. Compound **17** was identified as 10-O-demethybocconoline [17].

Compounds **16** and **18** were deduced to be isoarnottianamide and arnottianamide [5]. These two compounds were derivatives of benzophanthridine containing N-methylformamide group, whose mass spectrum a minor peak corresponding to the loss of HCONHCH_3_ was observed. Compounds **16** and **18** displayed the same protonated molecule ion at *m*/*z* 382.1291 (C_21_H_20_NO_6_^+^) at retention times of 16.8 and 17.5 min, respectively. Both of them produced the fragment ions at 364.1185 (C_20_H_20_NO_5_^+^) and 323.0913 (C_18_H_12_NO_3_^+^) resulted from the loss of H_2_O and HCONHCH_3_, respectively.

Besides skimmianine, six quinoline alkaloids (**5**, **6**, **9**, **12**, **14**, **15**) were detected. Compounds **5**, **6**, and **9** were reported for the first time in *Z. nitidum*.

Compounds (**6**, **12**, **14**, **15**) were furanquinoline alkanoids. For furanquinoline compounds containing vicinal methoxy groups at C7 and C8, the successive loss of H_2_O following CH_3_ was a typical characteristic [27]. The main fragment pattern of the other furanquinoline alkanoids was the loss of CH_3_, CO, and CO, the final loss of CO might come from phenolic hydroxyl or furan ring [32]. Compounds **6** and **12** displayed the same protonated molecule ion at *m*/*z* 246.0761 (C_13_H_12_NO_4_^+^) at retention times of 9.8 and 13.1 min, respectively. Compound **6** yielded the fragment ions at 231.0531 (C_12_H_9_NO_4_^+^), 213.0419 (C_12_H_7_NO_3_^+^) and 185.0470 (C_11_H_7_NO_2_^+^) via the consecutive loss of CH_3_, H_2_O and CO. The fragmentation pathway was like that of skimmianine, suggesting the existence of adjacent methoxy groups at C7 and C8. Compound **6** was identified as 4-hydroxy-7, 8-dimethoxyl-furanquinoline [27]. Compound **12** generated the fragment ions at 231.0531(C_12_H_9_NO_4_^+^), 216.0289 (C_11_H_6_NO_4_^+^), and 188.0327 (C_10_H_6_NO_3_^+^) due to the consecutive losses of CH_3_, CH_3_, and CO. The fragmentation pathway showed that compound **12** do not possess adjacent methoxy groups at C7 and C8. So, it might contain C7-OH or C8-OH. Because C7-OH is less polar than C8-OH, isomer with C7-OH must be eluted later. Comparing the retention time of two isomers with our previous published reference [27], compound **12** was speculated as the isomer with C7-OH. Thus, compound **12** was identified as haplopine [7].

Compound **14** displayed the protonated molecule ion at *m*/*z* 230.0817 (C_13_H_12_NO_3_^+^) at retention times of 15.8 min. The subsequent fragment ions at *m*/*z* 215.0575 (C_12_H_9_NO_3_^+^), 200.0341 (C_11_H_6_NO_3_^+^), 172.0391 (C_10_H_6_NO_2_^+^), 144.0403 (C_9_H_6_NO^+^), and 116.0499 (C_8_H_6_N^+^) were observed as a result of the losses of CH_3_, CH_3_, CO, CO and CO. Compound **14** was identified as γ-fagarine [16].

Compound **15** yielded the protonated molecule ion at *m*/*z* 200.0706 (C_12_H_10_NO_2_^+^) at retention times of 16.6 min. The fragment ions at *m*/*z* 185.0471 (C_11_H_7_NO_2_^+^), 157.0522 (C_10_H_7_NO^+^) and 129.0573 (C_9_H_7_N^+^) resulted from the consecutive losses of CH_3_, CH_3_ and CO. Compound **15** was tentatively identified as dictammine [9].

Compounds **5** and **9** eluted at 8.4 and 11.4 min, respectively, must contain dihydropyran ring with hydroxyl. Both shared the same protonated molecule ion at *m*/*z* 260.1287 (C_15_H_18_NO_3_^+^). These two compounds all produced the fragment ions corresponding to the loss of H_2_O and C_4_H_8_O. The loss of C_4_H_8_O were produced by RDA reaction of dihydropyran ring. Therefore, compounds **5** and **9** were identified as 3-hydroxy, 2, 2, 6-trimethyl-3, 4, 5, 6-tetrahydro-2H-pyrano [3, 2-c] quinoline 5-one and ribalinine, which had been reported to be isolated from Skimmia laureola Hook, a plant of family Rutaceae [33].

### 2.2. Quantitative Method Validation

After oral administration of *Z. nitidum* decoction, a total of 19 prototype alkaloids were identified. Considering the quality control component of *Z. nitidum* suggested by Chinese Pharmacopoeia 2015, content in *Z. nitidum*, plasma exposure level and the availability of reference standard, magnoflorine, α-allocryptopine, nitdine, chelerythrine, and skimmianine were selected to perform pharmacokinetic experiments by HPLC-MS/MS. However, the results of the preliminary experiment showed that nitidine and chelerythrine had poor absorption, as reported in the literatures [34,35]. Moreover, obvious interference was observed from endogenous material at the rentention times of nitidine and chelerythrine during the chromatographic separation, despite various sample preparation methods were applied. Finally, magnoflorine, α-allocryptopine, and skimmianine were selected for the further pharmacokinetic study. The corresponding quantification method using HPLC-MS/MS were developed.

**Figure 6:**
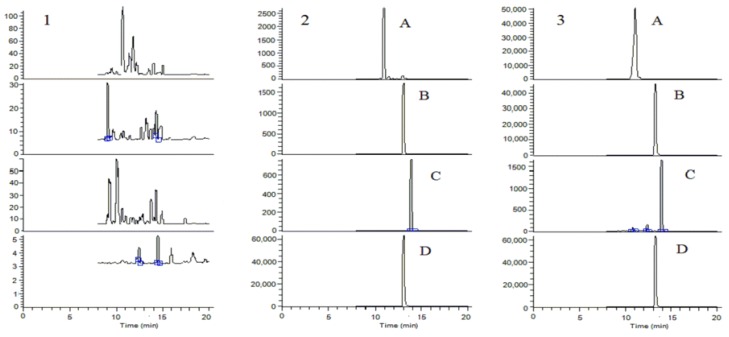
The typical SRM chromatograms. **1**. Blank plasma; **2**. Blank plasma spiked with three analytes at LLOQ level and IS; **3**. Plasma collected at 1 h after oral administration of *Z. nitidum* decoction. **A**. Magnoflorine, **B**. α-allocryptopine, **C**. Skimmianine and **D**. internal standard (IS).

The typical chromatograms of blank plasma, blank plasma spiked with three analytes and internal standard (IS), and plasma after oral administration of *Z. nitidum* decoction were shown in Figure 6. No obvious interference was observed from endogenous material at the rentention times of analytes and IS. As shown in Table 2, magnoflorine, α-allocryptopine, and skimmianine showed good linearity (r > 0.999) over the linear range. The lower limit of quantification (LLOQ) of magnoflorine, α-allocryptopine and skimmianine were 2, 2, and 0.5 ng/mL, respectively. The intra- and inter-day precision and accuracy were summarized in Table 3. All analytes displayed relative standard deviation (RSD%) below 11.23% and relative error (RE%) ranged from 8.05% to 11.23%, which were within the acceptable criteria. The extraction recovery of magnoflorine, α-allocryptopine and skimmianine were in the range of 89.87–98.32% and IS was 93.90%. The matrix effects of three analytes were in the range of 92.73–108.46% and IS was 93.88%. The stability of analytes under four storage conditions were assessed and the results were listed in Table 4. All analytes exhibited RSD% below 11.32% and RE% ranged from 10.00% to 12.53%, indicating that the analytes were stable. In conclusion, the developed method was validated and satisfactory for pharmacokinetic study.

### 2.3. Pharmacokinetic Study

**Figure 7:**
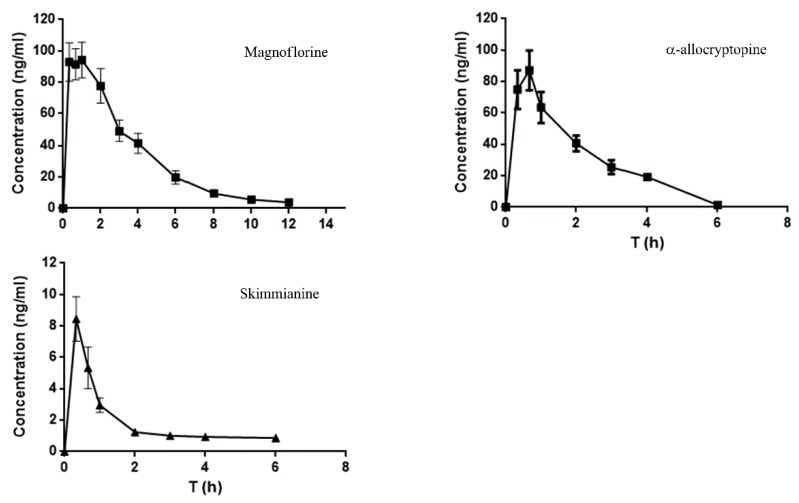
Plasma concentration-time curves of analytes.

The validated HPLC-MS/MS method was successfully applied for the pharmacokinetic study of magnoflorine, α-allocryptopine, and skimmianine in rat plasma after oral administration of *Z. nitidum* decoction. The plasma concentration-time curve was shown in Figure 7. The main pharmacokinetic parameters were processed by Drug and Statistics (DAS) 2.0 software and listed in Table 5.

As shown in Figure 7, the three components were absorbed rapidly after oral administration, with T_max_ ranged from 0.38 to 1.05 h. The C_max_ of magnoflorine and α-allocryptopine were about ten times higher than that of skimmianine. Combined with their contents in *Z. nitidum* decoction (6.7, 1.1 and 0.4 mg/mL), the absorption rate of α-allocryptopine might be the highest of these three alkaloids. The T_1/2_ of magnoflorine, α-allocryptopine and skimmianine were 3.24 ± 1.31, 0.78 ± 0.17 and 5.99 ± 1.62 h, respectively. The total exposure area under curve (AUC)_0–∞_ of magnoflorine was the largest of the three components. The relatively higher plasma concentration and AUC_0–∞_ indicated that magnoflorine and α-allocryptopine might have favorable drug-like properities.

Magnoflorine has been reported for its diverse pharmacological properties, such as anti-inflammatory, anti-bacteria and immunomodulatory effects [36,37,38,39]. Some of these properties might contributed to anti-inflammation and analgesic effect of *Z. nitidum* decoction in clinic use. Limited information is known about pharmacological properties of α-allocryptopine. Available studies indicate that α-allocryptopine possesses antiarrhythmic effects [40,41,42]. Skimmianine has been reported to possess anti-inflammatory and non-narcotic analgesic effects etc. [43,44,45], but its plasma concentration and AUC_0–∞_ was relatively lower in this study. Whether there exist synergistic action of them needs further investigation.

As for nitidine, due to poor absorption, its functional mechanism in vivo needs further study.

## 3. Experimental

### 3.1. Chemical, Reagents and Materials

The roots of *Z. nitidum* were collected from Guangdong, China, and authenticated by RT Zhan. Magnoflorine (No. 130611, purity 98.0%) and α-allocryptopine (No.150510, purity 98.0%) were purchased from Sichuan Victory Bio-Technology Ltd. (Chengdu, China) Co., nitidine chloride (No.110848, purity 98.0%) and chelerythrine (No.110718, purity 98.0%) were purchased from the National Institute for the Control of Pharmaceutical and Biological Product (Beijing, China), skimmianine (No.160106, purity 98.0%) was purchased from Shanghai yuanmu Bio-Technology Ltd. (Shanghai, China) Co. Acetonitrile and formic acid were of HPLC grade agents and obtained from Merck (Darmstadt, Germany). All other agents were of analytical grade and purchased from Guangzhou Chemical Reagent Factory (Guangzhou, China). Triple deionized water was prepared using a Milli-Q system (Millipore, Billerica, MA, USA). The HyperSep C18 solid-phase extraction (SPE) column (1000 mg, 6 mL) was purchased from Thermo Electron Corporation (Waltham, MA, USA).

### 3.2. Instrumentation and Analytical Conditions

#### 3.2.1. Qualitative Analysis

The UPLC analysis was performed on a Shimadzu Nexera UHPLC LC-30A system (Shimadzu Corporation, Tokyo, Japan). The separation was executed by an ODS column (Shimadzu, 2.0 mm i.d. × 100 mm, 1.9 μm) maintained at 35 °C. The mobile phase consisted of Solvent A (0.1% formic acid solution) and Solvent B (0.1% formic acid acetonitrile). The flow rate was 0.4 mL/min. The gradient program was as follows: 5–20% B at 0–10 min; 20–35% B at 10–15 min; 35–100% B at 15–20 min.

MS spectra were achieved on an AB SCIEX Triple TOF 5600 (AB Sciex Pte. Ltd., Singapore, Singapore) with electrospray ionization (ESI) source in positive mode. The following parameters of mass spectra were used: source temperature at 500 °C; nebulizer and heater gas pressure at 50 psi; curtain gas pressure at 40 psi; ion spray voltage at 5500 V; declustering potential at 100 V, collision energy 10 eV, and mass range 100–800 amu. The collision energy in “product ion” scan was set at 35 v with a collision energy spread of 10 eV. Data acquisition was controlled with AB SCIEX Analyst TF (Version 1.7) software (AB Sciex Pte. Ltd., Singapore, Singapore). Data processing was performed with Peakview (Version 2.0) software (AB Sciex Pte. Ltd., Singapore, Singapore).

#### 3.2.2. Quantitative Analysis

The HPLC analysis performed on a Surveyor plus HPLC system (Thermo Fisher Scientific, Waltham, MA, USA). The separation was executed by a Thermo Accucore aq C18 column (100 × 2.1 mm, 2.6 μm) (Thermo Fisher Scientific, Waltham, MA, USA) maintained at 30 °C. The mobile phase consisted of Solvent A (0.1% formic acid solution) and Solvent B (0.1% formic acid acetonitrile). The flow rate was 0.3 mL/min. The gradient program was as follows: 5–10% B at 0–5 min; 10–100% B at 5–15 min; 100–5% B at 15–15.1 min; 5% B at 15–20 min.

MS data were achieved on a triple-quadrupole mass spectrometer: TSQ Quantum Access (Thermo Fisher Scientific, Waltham, MA, USA) with electrospray ionization (ESI) source in positive mode. The instrument parameters were as follows: spray voltage at 3000 V, sheath gas and auxiliary gas with a flow of 30 and 5 arbitrary units, capillary temperature at 350 °C, collision gas pressure at 1.0 mTorr, skimmer offset at 2 V. The selected reaction monitoring (SRM) transitions used for MS analysis were as follows: *m*/*z* 342→297 for magnoflorine, 370→188 for α-allocryptopine, 260→227 for skimmianine, 356→192 for IS; the collision energies at 15 eV for magnoflorine, 30 eV for α-allocryptopine, skimmianine and IS. The data acquisition and processing was performed with Xcalibur 2.0 software (Thermo Fisher Scientific, Waltham, MA, USA).

### 3.3. Preparation of Samples

#### 3.3.1. Preparation of *Z. nitidum* Decoction

100 g of the roots of *Z. nitidum* were weighed and decocted with 1.2 L of water for 3 h. The filtrate was collected and residue was decocted in 1.2 L of water for 2 h again. Subsequently, the filtrates from each decoction were combined and concentrated to 50 mL. The contents of magnoflorine, α-allocryptopine, nitidine, chelerythrine, and skimmianine in *Z. nitidum* decoction were 6.7, 1.1, 1.8, 3.5, and 0.4 mg/mL, respectively.

#### 3.3.2. Preparation of Plasma Samples

##### Qualitative Analysis

The 2 mL plasma sample was loaded on a pretreated SPE column which was eluted with 20 mL methanol followed by 20 mL water. After being washed off by 6 mL of water, the cartridge was eluted using 6 mL methanol. The methanol eluting was evaporated to dryness at 35 °C in vacuum using SpeedVac Concentration (Savant SPD 1010, Thermo scientific). The residue was reconstituted in 300 μL acetonitrile and water (50:50, *v*/*v*) and centrifuged at 13,000 rpm (15,493× *g*) for 15 min.

##### Quantitative Analysis

100 μL plasma sample, 10 μL IS solution (200 ng/mL) and 300 μL acetonitrile were added to a 1.5 mL eppendorf tube and vortex-mixed for 5 min, then centrifuged at 13,000 (15,493× *g*) rpm for 10 min. The supernatant was transferred into another eppendorf tube and evaporated to dryness at 35 °C in vacuum using SpeedVac Concentration ((RVC 2-18, Christ). The residue was reconstituted in 100 μL acetonitrile and water (50:50, *v*/*v*) and centrifuged at 13,000 rpm (15,493× *g*) for 15 min.

#### 3.3.3. Calibration Samples and Quality Control Samples

The stock solutions of magnoflorine, α-allocryptopine and skimmianine were prepared in acetonitrile at 1.06, 1.02, and 0.98 mg/mL, respectively. A serious of working solutions were obtained by diluting with acetonitrile. The IS solution was prepared at a concentration of 200 ng/mL in acetonitrile.

The calibration working solution were prepared by spiking the working solution into blank plasma to obtain concentrations ranges from 2–200 ng/mL for magnoflorine and α-allocryptopine, from 0.5–50 ng/mL for skimmianine. Quality control (QC) samples were obtained at 5, 50, 160 ng/mL for magnoflorine and α-allocryptopine, at 1, 10, 32 ng/mL for skimmianine.

### 3.4. Method Validation

Validation of the analytical method was assessed on selectivity, linearity, sensitivity, accuracy, precision, recovery, matrix effect, and stability according to Pharmacopoeia of the People’s Republic of China 2015 guidelines.

The selectivity was assessed by analyzing the chromatograms of blank plasma of six different rats, a blank plasma with magnoflorine, α-allocryptopine, skimmianine and IS, and a plasma after dose. The linearity was determined by plotting the peak areas ratios (y) of each analyte to IS against the concentrations, and evaluated by least-squares linear regression. The lower limit of quantification (LLOQ) was defined as the lowest concentration point of the calibration curve (S/N > 10) with the accuracy within ±20% and precision lower than 20%. The accuracy and precision were evaluated by analyzing the six replicate QC samples on the same day (intra-day) and three consecutive days. The accuracy and precision were expressed as relative error (RE%) and relative standard deviation (RSD%), respectively. The extraction recovery was determined by comparing peak areas of the extracted QC samples with those of post-extracted spiked samples. The matrix effects was measured by calculated the analytes peak area ratios of post-extracted spiked samples to those of pure work solution. The stability was investigated by analyzing samples stability under diverse storage conditions: three freeze-thaw cycles, 8 h at 25 °C, −80 °C for 40 days and in autosampler for 24 h.

### 3.5. Animal Experiments

Male Sprague–Dawley rats (250 ± 20 g) used in this study were provided by the Experimental Animal Center of Guangzhou University of Chinese Medicine. The laboratory animal license number is SCXK 2013-0020. These animals were maintained in an air-conditioned animal facility at 23 ± 2 °C, with a humidity of 55% ± 5% and a 12 h light/dark cycle for 5 days before use. The rats had free access to water and a standard diet. Animal welfare and experimental procedures were strictly in accordance with the guidelines of the Committee on the Care and Use of Laboratory Animals in China and the related ethical regulations of Guangzhou University of Chinese Medicine.

For profile study, twelve rats were randomly divided into 2 group, blank control and experimental groups. Before administration, the rats were fasted for 12 h but allowed water ad libitum. *Z. nitidum* decoction was orally administered to experimental group at a dose of 15 mL.kg^−1^ (15 mL decoction equal to 30 g crude drug) body weight, while distilled water was orally administered to control group. The rats of experimental group were anesthetized at 0.5 h, 1 h, 2 h, 3 h, 4 h, and 6 h after dose, respectively. The blood samples were collected from aorta abdominalis in heparinized tube. All blood samples were then centrifuged at 3500 rpm (1274× *g*) for 15 min at 4 °C. Blank plasma samples were prepared following the same procedures. All samples were stored at −80 °C.

For pharmacokinetic study, six rats were orally administered *Z. nitidum* decoction at a dose of 5.4 mL kg^−1^ (5.4 mL decoction equal to 10.8 g crude drug) body weight. The blood samples were collected from orbital vein before dose and 0.17, 0.33, 0.67, 1, 2, 3, 4, 6, 8, 10, 12, and 24 after dose. The blood samples were then centrifuged at 3500 rpm (1274× *g*) for 15 min at 4 °C. The samples were stored at −80 °C. Data analysis was performed by Drug and Statistics (DAS) 2.0 software (Mathematical Pharmacology Professional Committee of China, Shanghai, China).

## 4. Conclusions

In this study, the UPLC-Q-TOF-MS/MS method was used to identify the absorbed alkaloids *in vivo* after oral administration *Z. nitidum* decoction for the first time. The fragmentation pathway of magnoflorine, α-allocryptopine and nitidine was proposed, and a total of 19 alkaloids were exactly or tentatively identified in rat plasma after dose, including 2 aporphinoid, 3 protopine, 7 benzophenanthrindine, 7 quinoline alkaloids. Among them, five constituents were reported for the first time in *Z. nitidum*. In addition, a HPLC-MS/MS method was developed to simultaneous determination of three main absorbed components, including magnoflorine, α-allocryptopine, and skimmianine for the first time. This HPLC-MS/MS method was applied to pharmacokinetic study after oral administration *Z. nitidum* decoction. These results would be helpful for a better understanding about material basis and function mechanism of *Z. nitidum* decoction, and also provided important information for the quality control and further pharmacological study.

## Figures and Tables

**Table 1:** MS data and identification results of the alkaloids in rat plasma after oral administration of *Z. nitidum* decoction.

NO.	RT (min)	Mass Found	Error (ppm)	Selected ion	Formula	MS^2^ Ions	Identification	Types
**1**	3.2 ^*^	518.2016	0.3	[M + H]^+^	C_26_H_32_NO_10_	356.1491, 338.1389, 188.0704	glucothalictrisine/glucohunnemannine	protopine
**2**	4.2	342.1702	−1	[M]^+^	C_20_H_24_NO_4_	297.1111, 282.0876, 265.0848, 237.0900	magnoflorine	aporphine
**3**	5.3 ^*^	356.1494	−1.2	[M + H]^+^	C_20_H_22_NO_5_	338.1389, 275.0698, 206.0809, 188.0809	thalictrisine/hunnemannine	protopine
**4**	8.1	370.1664	0.8	[M + H]^+^	C_21_H_23_NO_5_	352.1555, 206.0813 189.0773, 188.0704	α-allocryptopine	protopine
**5**	8.4 ^*^	260.1284	1.1	[M + H]^+^	C_15_H_17_NO_3_	242.1180, 188.0709, 176.0706, 134.0609	2.2,6-trimethy-3,4,5,6-tetrahydro-2H-pyrano[3,2-c]quinoline	quinoline
**6**	9.8 ^*^	246.0761	0.2	[M + H]^+^	C_13_H_12_NO_4_	231.0526, 213.0419, 185.0471	4-hydroxy-7,8-dimethoxy-furanquinoline	quinoline
**7**	9.9	334.1072	−2	[M]^+^	C_20_H_16_NO_4_	319.0831, 291.0886, 276.0663	isofagaridine	benzophenanthrine
**8**	11.3	348.1239	0.7	[M]^+^	C_21_H_18_NO_4_	332.0934, 304.0985, 290.0806	nitidine	benzophenanthrine
**9**	11.4 ^*^	260.1283	0.9	[M + H]^+^	C_15_H_17_NO_3_	242.1174, 188.0709, 176.0706, 134.0609	ribalinine	quinoline
**10**	11.6	348.1231	0.5	[M]^+^	C_21_H_18_NO_4_	332.0923, 304.0975, 290.0817	chelerythrine	benzophenanthrine
**11**	12.2	276.0656	0.5	[M + H]^+^	C_17_H_9_NO_3_	248.0698, 218.0594 190.0636	liriodenine	aporphine
**12**	13.1	246.0759	−0.5	[M + H]^+^	C_13_H_12_NO_4_	231.0526, 216.0286, 188.0346	haplopine	quinoline
**13**	15.5	260.0916	0.9	[M + H]^+^	C_14_H_13_NO_4_	245.0682, 227.0575, 199.0627	skimmianine	quinoline
**14**	15.8	230.0809	−0.9	[M + H]^+^	C_13_H_11_NO_3_	230.0809, 215.0809, 186.0539, 172.0573	γ-fagarine	quinoline
**15**	16.6	200.0703	−1.5	[M + H]^+^	C_12_H_9_NO_2_	185.0471,129.0579	dictamine	quinoline
**16**	16.8	382.1287	0.4	[M + H]^+^	C_21_H_19_NO_6_	364.1177, 354.1321, 349.0947, 323.0913, 292.0724	isoarnottianamide	benzophenanthrine
**17**	17.4	366.1340	1	[M + H]^+^	C_21_H_19_NO_5_	348.1232, 333.0983, 320.0920, 305.0700, 292.0739, 275.0700	O-demethyl-becconoline	benzophenanthrine
**18**	17.5	382.1288	0.7	[M + H]^+^	C_21_H_19_NO_6_	364.1179, 354.1334, 339.1098, 336.1225, 292.0718	arnottianamide	benzophenanthrine
**19**	19.1	334.1076	0.8	[M + H]^+^	C_20_H_16_NO_4_	319.0848, 318.0760, 290.0840	Norchelerythrine	benzophenanthrine

RT mean retention time, and ^*^ mean that the compound was reported for the first time in *Z. nitidum*.

**Table 2:** Regression equation, correlation coefficients, linear range and lower limit of quantification (LLOQ) of analytes.

Analyte	Linear Regression Equation	r	Linear Range (ng/mL)	LLOQ		
				Concentration (ng/mL)	RSD (%)	RE (%)
magnoflorine	y = 0.011x + 0.018	0.9990	2–200	2	8.72	−6.88
α-allocryptopine	y =0.012x + 0.010	0.9995	2–200	2	13.04	−7.25
skimmianine	y = 0.014x − 0.010	0.9996	0.5–50	0.5	14.81	5.63

**Table 3:** Precision, accuracy, extraction recovery and matrix effect of analytes in rat plasma (*n* = 6).

Analyte	Concentration (ng/mL)	Intraday		Interday		Extraction Recovery (Mean ± SD, %)	Matrix Effect (Mean ± SD, %)	Related Matrix Effect (RSD, %)
		RSD (%)	RE (%)	RSD (%)	RE (%)			
magnoflorine	5	4.22	−8.05	10.0	11.03	89.87 ± 7.75	108.46 ± 9.4	6.51
	50	2.89	5.62	8.12	−3.45	90.52 ± 3.16	98.91 ± 5.59	4.76
	160	6.78	3.16	5.26	6.64	95.57 ± 3.82	94.47 ± 7.18	5.43
α-allocryptopine	5	1.83	9.33	3.96	7.56	92.68 ± 5.38	102.38 ± 8.43	8.37
	50	3.75	−3.87	5.77	−1.57	96.08 ± 6.77	99.32 ± 4.35	7.12
	160	2.23	1.82	7.02	−8.02	94.30 ± 4.16	93.82 ± 5.63	5.56
skimmianine	1	2.62	4.41	4.01	6.33	93.41 ± 4.41	101.10 ± 6.42	6.39
	10	2.84	6.51	11.23	−7.75	98.32 ± 6.47	99.39 ± 5.84	8.41
	32	3.56	4.97	2.65	4.39	94.06 ± 3.56	92.73 ± 5.52	5.17

SD: standard deviation.

**Table 4:** Stability of analytes in rat plasma (*n* = 6).

Analyte	Concentration (ng/mL)	Three Freeze-Thaw Cycle		8 h at Room Temperature		24 h at 4 °C		40 Days at = −80 °C	
		RSD (%)	RE (%)	RSD (%)	RE (%)	RSD (%)	RE (%)	RSD (%)	RE (%)
magnoflorine	5	7.53	−10.00	8.25	−5.14	4.46	−5.59	6.43	10.17
	50	4.58	11.03	3.26	6.87	3.81	9.24	3.68	−8.92
	160	5.54	9.16	1.53	5.74	9.95	4.61	4.15	5.74
α-allocryptopine	5	8.61	8.97	6.55	12.53	2.07	2.99	5.88	8.59
	50	6.62	−5.58	3.68	−7.13	6.14	−6.21	3.71	−4.82
	160	1.19	4.24	1.59	3.27	1.26	7.75	2.73	−6.91
skimmianine	1	9.22	10.43	11.06	−9.41	8.18	9.47	8.57	9.69
	10	8.51	−5.95	10.44	6.62	11.32	−4.39	9.92	−6.48
	32	7.13	−9.01	8.43	9.51	4.31	−1.09	8.01	−4.07

**Table 5:** Pharmacokinetic parameters of analytes in rats after oral administration.

Parameters	Analytes (Mean ± SD, *n* = 6)		
	Magnoflorine	α-ALlocryptopine	Skimmianine
Cmax (ng/mL)	112.69 ± 18.79	100.28 ± 49.91	8.91 ± 1.89
Tmax (h)	1.05 ± 0.71	0.47 ± 0.13	0.38 ± 0.12
T1/2 (h)	3.24 ± 1.31	0.78 ± 0.17	5.99 ± 1.62
AUC0-t (h*ng/mL)	408.13 ± 91.34	180.361 ± 96.32	11.08 ± 2.02
AUC0-∞ (h*ng/mL)	437.99 ± 106.29	186.41 ± 98.68	17.05 ± 6.32

AUC: area under curve.

## Supplementary Materials

The following are available online.
