# Supplementary material for: Profiling and Pharmacokinetic Studies of Alkaloids in Rats After Oral Administration of Zanthoxylum nitidum Decoction by UPLC-Q-TOF-MS/MS and HPLC-MS/MS
Source: Molecules. 2019 Feb 7;24(3):585. doi: 10.3390/molecules24030585 (PMC6384758; doi:10.3390/molecules24030585)
Supplement: Supplementary file 1 [file molecules-24-00585-s001.zip › Supplementary File/Table S1.pdf]

Table S1 The information of the alkaloids isolated from the *Zanthoxylu nitidum*

No.	Name	Formula	Selected ion	Exact mass	Type	Literature
1	magnoflorine	$C_{20}H_{24}NO_4$	M	342.1705	aporphine	[4]
2	liriodenine	$C_{17}H_9NO_3$	M+H	276.0655	aporphine	[5-9]
3	isotembetarine	$C_{20}H_{26}NO_4$	M	344.1862	benzylisoquinoline	[10]
4	α-allocryptopine	$C_{21}H_{23}NO_5$	M+H	370.1649	protopine	[5,8,11]
5	coptisine	$C_{19}H_{14}NO_4$	M	320.0923	protoberberine	[12]
6	protoberberrubine	$C_{19}H_{16}NO_4$	M	322.1079	protoberberine	[12]
7	nitidine	$C_{21}H_{18}NO_4$	M	348.1236	benzophenanthrine	[5,8,11,13-15]
8	chelerythrine	$C_{21}H_{18}NO_4$	M	348.1236	benzophenanthrine	[5,13,14]
9	sanguinarine	$C_{20}H_{14}NO_4$	M	332.0923	benzophenanthrine	[12]
10	isofgaridine	$C_{20}H_{16}NO_4$	M	334.1079	benzophenanthrine	[14]
11	decarine	$C_{19}H_{13}NO_4$	M+H	320.0917	benzophenanthrine	[5,6]
12	N-norchelerythrine	$C_{20}H_{16}NO_4$	M+H	334.1079	benzophenanthrine	[5-8,13]
13	rhoifoline B	$C_{21}H_{17}NO_5$	M+H	364.1185	benzophenanthrine	[16]
14	oxynitidine	$C_{21}H_{17}NO_5$	M+H	364.1180	benzophenanthrine	[5,7,11,13]
15	oxychelerythrine	$C_{21}H_{17}NO_5$	M+H	364.1180	benzophenanthrine	[5,13]
16	oxyterihanine	$C_{20}H_{16}NO_5$	M+H	350.1028	benzophenanthrine	[5]
17	oxyavicine	$C_{20}H_{13}NO_5$	M+H	348.0866	benzophenanthrine	[7,15]
18	6-hydroxymethyldihydronitidine	$C_{22}H_{21}NO_5$	M+H	380.1498	benzophenanthrine	[16]
19	bocconoline	$C_{22}H_{21}NO_5$	M+H	380.1498	benzophenanthrine	[5,16]
20	O-demethyl- bocconoline	$C_{21}H_{19}NO_5$	M+H	366.1336	benzophenanthrine	[17]
21	dihydronitidine	$C_{21}H_{19}NO_4$	M+H	350.1387	benzophenanthrine	[3,8,11]
22	dihydrochelerythrine	$C_{21}H_{19}NO_4$	M+H	350.1387	benzophenanthrine	[5-8,15]
23	6-methoxynitidine	$C_{22}H_{21}NO_5$	M+H	380.1492	benzophenanthrine	[18]
24	6-methoxychelerythrine	$C_{22}H_{21}NO_5$	M+H	380.1492	benzophenanthrine	[3,5,15]
25	6-ethoxychelerythrine	$C_{23}H_{23}NO_5$	M+H	394.1654	benzophenanthrine	[5,13]
25	(R)-6-[(R)-1-hydroxyethyl]dihydrochelerythrine (1)	$C_{23}H_{23}NO_5$	M+H	394.1654	benzophenanthrine	[15]
27	6-methoxynorchelerythrine	$C_{21}H_{17}NO_5$	M+H	364.1185	benzophenanthrine	[15]
28	6-hydroxydihydrochelerythrine	$C_{21}H_{19}NO5$	M+H	366.1341	benzophenanthrine	[15]
29	6-acetonyldihydronitidine	$C_{24}H_{23}NO_5$	M+H	406.1654	benzophenanthrine	[8]

30	6-acetonyldihydrochelerythrine	$C_{24}H_{23}NO_5$	M+H	406.1654	benzophenanthrine	[8]
31	6-acetonyldihydroavicine	$C_{23}H_{19}NO_5$	M+H	390.1341	benzophenanthrine	[8]
32	6-methoxyisodecarine	$C_{20}H_{15}NO_5$	M+H	350.1028	benzophenanthrine	[19]
33	6-methoxysanguinarine	$C_{21}H_{17}NO_5$	M+H	364.1185	benzophenanthrine	[19]
34	zanthomuurolanine	$C_{37}H_{45}NO_5$	M+H	584.3370	benzophenanthrine	[20]
35	epi-zanthomuurolanine	$C_{37}H_{45}NO_5$	M+H	584.3370	benzophenanthrine	[20]
36	zanthocadinanines A	$C_{37}H_{45}NO_5$	M+H	584.3370	benzophenanthrine	[20]
37	zanthocadinanines B	$C_{37}H_{45}NO_5$	M+H	584.3370	benzophenanthrine	[20]
38	epi-zanthocadinanine B	$C_{37}H_{45}NO_5$	M+H	584.3370	benzophenanthrine	[20]
39	isoarnottianamide	$C_{21}H_{19}NO_6$	M+H	382.1285	benzophenanthrine	[5]
40	arnottianamide	$C_{21}H_{19}NO_6$	M+H	382.1285	benzophenanthrine	[5,6,8]
41	integriamide	$C_{20}H_{15}NO6$	M+H	366.0972	benzophenanthrine	[5]
42	skimmianine	$C_{14}H_{13}NO_4$	M+H	260.0917	quinoline	[5,7-9,11,13,16,18]
43	haplopine	$C_{13}H_{11}NO_4$	M+H	246.0761	quinoline	[7]
44	dictamnine	$C_{12}H_9NO_2$	M+H	200.0706	quinoline	[9,16,18]
45	γ-fagarine	$C_{13}H_{11}NO_3$	M+H	230.0812	quinoline	[16,18]
46	5-methoxydictamnine	$C_{13}H_{11}NO_3$	M+H	230.0812	quinoline	[18]
47	robustine	$C_{12}H_9NO_3$	M+H	216.0661	quinoline	[16]
48	zanthobungeanine	$C_{16}H_{17}NO_3$	M+H	272.1287	quinoline	[9]
49	zanthodioline	$C_{16}H_{19}NO_5$	M+H	306.1136	quinoline	[9]
50	edulinine	$C_{16}H_{21}NO_4$	M+H	292.1549	quinoline	[8]
51	N-methylflindersine	$C_{15}H_{15}NO_2$	M+H	242.1181	quinoline	[9]
52	4-methoxy-(-1)-methyl-2-quinolone	$C_{11}H_{11}NO_2$	M+H	190.0868	quinoline	[16]
